# Attentional and cognitive monitoring brain networks in long-term meditators depend on meditation states and expertise

**DOI:** 10.1038/s41598-021-84325-3

**Published:** 2021-03-01

**Authors:** Juliana Yordanova, Vasil Kolev, Valentina Nicolardi, Luca Simione, Federica Mauro, Patrizia Garberi, Antonino Raffone, Peter Malinowski

**Affiliations:** 1 Institute of Neurobiology, Bulgarian Academy of Sciences, Acad. G. Bonchev str., bl. 23, 1113 Sofia, Bulgaria; 2 Department of Psychology, Sapienza University of Rome, Rome, Italy; 3 Social and Cognitive Neurosciences Laboratory, IRCCS, Santa Lucia Foundation, Rome, Italy; 4 Institute of Cognitive Sciences and Technologies, CNR, Rome, Italy; 5 School of Buddhist Studies, Philosophy and Comparative Religions, Nalanda University, Rajgir, India; 6 School of Psychology, Research Centre for Brain and Behaviour, Liverpool John Moores University (LJMU), Liverpool, UK

**Keywords:** Cognitive control, Consciousness, Cognitive neuroscience, Attention

## Abstract

Meditation practice is suggested to engage training of cognitive control systems in the brain. To evaluate the functional involvement of attentional and cognitive monitoring processes during meditation, the present study analysed the electroencephalographic synchronization of fronto-parietal (FP) and medial-frontal (MF) brain networks in highly experienced meditators during different meditation states (focused attention, open monitoring and loving kindness meditation). The aim was to assess whether and how the connectivity patterns of FP and MF networks are modulated by meditation style and expertise. Compared to novice meditators, (1) highly experienced meditators exhibited a strong theta synchronization of both FP and MF networks in left parietal regions in all mediation styles, and (2) only the connectivity of lateralized beta MF networks differentiated meditation styles. The connectivity of intra-hemispheric theta FP networks depended non-linearly on meditation expertise, with opposite expertise-dependent patterns found in the left and the right hemisphere. In contrast, inter-hemispheric FP connectivity in faster frequency bands (fast alpha and beta) increased linearly as a function of expertise. The results confirm that executive control systems play a major role in maintaining states of meditation. The distinctive lateralized involvement of FP and MF networks appears to represent a major functional mechanism that supports both generic and style-specific meditation states. The observed expertise-dependent effects suggest that functional plasticity within executive control networks may underpin the emergence of unique meditation states in expert meditators.

## Introduction

Meditation has become a popular psychological tool, used in clinical and non-clinical contexts alike^1–5^. Accordingly, the neural substrates of meditation have attracted increasing research interest^6^. Such research is highly relevant, because a granular understanding of the distinct brain mechanisms that support meditation would enable utilizing meditation practices in an informed way and optimise their targeted implementation^7–11^.

One central neurophysiologic issue is whether meditation is a generic (or unique) brain state which can be cultivated by different meditation styles or whether brain states in meditation are profoundly different depending on the specific mental skills practiced in each style. In a previous study^12^, we addressed this question by analysing brain connectivity patterns in highly experienced meditators during three commonly used types of meditation: focused attention meditation (FAM), open monitoring meditation (OMM) and loving kindness meditation (LKM). We showed that brain states during these three meditation states share generic patterns that are substantially different from rest. In addition, we identified connectivity patterns that were specific to each meditation state.

The aim of the current study was to unpack the involved processes further by zooming in on the role of cognitive brain networks. Given the growing evidence that brain mechanisms of cognitive control are involved in meditation^6,13–16^, we surmise that training-induced neuroplasticity of these networks may contribute to generic brain states of meditation as well as to specific states that depends on the type of meditation^7,17^. Towards this end, we re-analysed our previous data set^12^ in the following directions: (1) we used FAM, OMM and LKM as three representative meditation states that differentially engage specific cognitive functions, (2) we explored the involvement of relevant executive control networks by analysing their synchronization patterns, and (3) we examined experienced meditators with a broad range of accumulated lifetime meditation practice to estimate the potential influence of meditation expertise on the involvement of executive networks.

### Meditation types

To address the question how executive control networks are involved in regulating cognitive processes in different meditation states, we focused on FAM, OMM and LKM^12^. Popular classification systems of meditation that integrate traditional understanding of meditation with psychological and neuro-functional perspectives single out these three meditation types as prominent meditation exemplars^6,18–21^.

FAM entails voluntarily focusing attention on a chosen object in a sustained fashion. In this context “objects” denotes physical objects in the external world, bodily sensations, such as the breath, but also mental objects, including thoughts, emotions or imagined visual forms. Focusing and sustaining attention on an object requires monitoring the focus of attention, detecting distraction, disengaging attention from the source of distraction, and (re)directing and engaging attention to the intended object^6,15^. Hence, the neuro-functional prediction is that specific neural systems in the human brain associated with selective attention, sustaining attention and conflict monitoring are involved in inducing and maintaining the state of FAM^6,13^.

OMM involves non-reactively monitoring experience from moment to moment without maintaining an explicit attentional focus on a specific object^6^. The aim of OMM is to cultivate meta-awareness of momentary sensations, thoughts and feelings, an ability closely linked to mindfulness^14,15,19,22,23^. Thus, OMM may primarily rely on brain systems that control and monitor cognitive states and regulate mind wandering, rather than on systems that establish and sustain object-focused attention^14^.

Whereas FAM and OMM cultivate distinct states of attention and meta-cognitive awareness, LKM aims to enhance prosocial and empathetic feelings, attitudes, and intentions^19^. This is achieved by cultivating acceptance of self and others, and the intention (motivation) toward wellbeing and happiness of self and others^5,24^. LKM shares some attributes with both FAM and OMM, such as top-down regulation of mind wandering, and the activation and maintenance of goal representations related to the meditation. Moreover, some aspects of LKM require object focus (e.g., focus on the mental representation of other people), resembling some aspects of FAM, whereas advanced forms of non-referential LKM bear similarity to OMM^6,25^. Therefore, it is plausible to expect that cognitive control, attentional and monitoring networks that are involved in FAM and OMM also play a role during LKM.

In the present study, we explored whether FAM, OMM and LKM would induce a common versus a differential involvement of attentional and cognitive monitoring brain systems, in line with the predicted differential involvement of these mental processes. Finding the same pattern across all three forms of meditation would indicate the activation of common executive processes in meditation, irrespective of the style, such as those supporting the intention or goal to perform the meditation task, attention to the present moment, sustained meta-awareness, regulation of mind wandering, and mental (executive) effort. By contrast, different patterns of activation of the attention and cognitive monitoring systems during each style of meditation would confirm the specificity of mental processes involved in meditation.

### Executive control networks

Importantly, the cognitive mechanisms playing a role in FAM, OMM and LKM—attention regulation, cognitive control, cognitive monitoring, and meta-awareness—are associated with anatomically and functionally dissociable systems in the brain, primarily the fronto-parietal and medial frontal networks^26,27^.

#### Fronto-parietal networks

From a functional point of view, fronto-parietal (FP) networks have been associated with spatial attention and orienting of attention. The dorsal FP networks are involved in the control of spatial and feature-based attention and in stimulus–response mapping, while the right-lateralized ventral FP network is linked to reorienting of attention to unexpected but behaviourally relevant events^26,28,29^. FP networks also subserve attention-based conscious perception^28,30–32^, cognitive control and working memory^33–37^, with prominent activations found in the right hemisphere^38^. Hence, the dorsal and ventral FP networks are essentially recognized as the neural substrate of focused attention and attention re-allocation^29,39^. Major operating frequencies of FP networks have been established for both slow and fast-frequency EEG ranges, in particular theta, alpha and beta frequency bands^40–42^.

#### Medial frontal network

Specific neuro-functional substrates have also been identified for a cognitive control and monitoring system in the medial frontal brain including the anterior cingulate cortex and medial frontal (MF) regions, brain areas that are linked to behavioural adaptation and control^27,43–46^. The major operating frequency of this monitoring system appears to be in the theta frequency band: medial frontal theta oscillations have been attributed a major coordinating function within the executive control system^47–49^. Specifically, theta-synchronizations between anterior cingulate cortex (ACC)/mid-frontal cortical areas and the basal ganglia, nucleus accumbens and prefrontal cortical areas have been associated with planning, strategic readjustment and adaptation^48^, as well as key elements of executive control–conflict processing, detection of errors, inhibition, and performance monitoring^43,49–51^.

To shed light on the connectivity patterns within FP and MF networks during meditation, we recorded 64-channel electroencephalographic (EEG) activity and calculated the spatial synchronization of EEG oscillations from different frequency bands. Patterns of spatial EEG synchronization were explored using the imaginary part of coherence (*ICoh*), a refined measure of connectivity^52^, after spatially enhancing the EEG signals.

### Meditation expertise

In the current study, highly experienced meditators were involved. Working with these virtuoso meditators offers a high degree of certainty that our participants are able to voluntarily enter and sustain the targeted mental states. Furthermore, we expected meditation training to be accompanied by neuroplastic and neurofunctional changes of relevant brain networks similar to other types of skill training. Including meditation experts with high levels of expertise may thus indicate how regular meditation practice shapes the dynamics of conventional brain FP and MF systems.

In summary, the aim of the present study was to characterize the functional connectivity of FP and MF networks in FAM, OMM and LKM in long-term practitioners.

## Materials and methods

### Participants

The sample of highly experienced meditators involved in our previous study^12^ was used to explore the role of cognitive control networks in meditation states. We studied 22 healthy right-handed volunteers (mean age = 44.2 years, SD = 9.9 years, age range 26–70 years, 4 females) who did not report any history of somatic disorders or neurological diseases. The participants were monks, nuns and novice practitioners residing at Amaravati Buddhist Monastery, in Southern England, or at Santacittarama Monastery, in Central Italy. Meditation practices at both monasteries are aligned with the Thai Forest Theravada Buddhist tradition, which is well established in the West. In this tradition, meditators practice FAM (Śamatha), OMM (Vipassana) and LKM (Metta) meditation forms in a balanced way, often in integrated sessions, including silent meditation retreats (at least 3 months per year). We estimated the meditation expertise of participants in hours, combining self-reported practice time before and during their monastic life. In this tradition, the monks, nuns, and novice practitioners typically practice 2 h per day with the monastery community, with regular more intensive practice during retreats (with several meditation sittings during the 3-month Winter retreat and other retreats). As suggested by the abbots of the monasteries, we estimated an average of 100 h of practice per month during monastic life, with a balance of FAM, OMM and LKM facets of meditation. The lifetime duration of meditation practice of the participants was estimated as a mean value = 19,358 h (SE = 3164), range 900–50,600 h. In line with the literature, and although characterized by intrinsic methodological problems, this estimation was based on an accurate recollection by the monastics, which was performed during several days before the experiment, by taking into account the average duration of daily practice over life periods with a consistent practice, as well as the average duration of daily practice during retreat periods and the number of retreat days. Furthermore, such recollection was facilitated by the practice regularities in the monastic life, both in terms of daily meditation sessions and intensive practice during retreats with established durations and routines over the years. The estimation of lifetime meditation practice was given by the sum of the hours of meditation practice during monastic life and the hours of comparable meditation practice before entering monastic life.

The study received approval by the Research Ethics Committee at Sapienza University of Rome, Italy. Prior to engaging with the study, all participants gave informed consent in line with the Declaration of Helsinki.

### Experimental design

As in^12^, participants had to perform a non-meditative rest condition (REST) and three meditation conditions: FAM, OMM and LKM. They were verbally instructed to switch between conditions. The instructions for the four conditions, which were written together with the abbot of Amaravati Monastery, the internationally recognized teacher Ajahn Amaro, were as follows:

*Rest*: “Rest in a non-meditative relaxed state, without falling in sleep, while allowing any spontaneous thoughts and feelings to arise and unfold in the field of experience”.

*Focused attention (Śamatha) meditation: “*Sustain the focus of attention on breath sensations, such as at the nostrils, noticing readily and with acceptance any arising distraction, such as on thoughts or stimuli, and in case of detected distraction, return readily and gently to focus attention on the breath sensations”.

*Open monitoring (awareness) meditation:* “With an open receptive awareness, observe the contents of experience as they arise, change and fade from moment to moment, without restrictions or judgments—such contents including breath and body sensations, sensations arising from contact with external stimuli, feelings and thoughts”.

*Loving kindness (Metta) meditation:* “Generate and sustain *metta*, acceptance and friendliness towards yourself and the experience in the present moment, as well as towards any being, in any state or condition”.

### EEG recordings and processing

*Procedure:* EEG was recorded with eyes closed in blocks of approximately 2.5–3 min each, repeating the sequence REST, FAM, OMM, LKM twice^12^. Thus, a total of approximately 5–6 min (2 blocks × 2.5–3 min) of data were recorded for each condition. EEG was acquired by a mobile wireless system (Cognionics; https://www.cognionics.net/mobile-128) using an electrode cap with 64 active Ag/AgCl electrodes located in accordance with the extended international 10/20 system and referenced to linked mastoids. Electrode impedances were kept below 10 kOhm and EEG signals were collected at a sampling rate of 500 Hz (resampled off-line to 250 Hz for data analysis). The experiment was conducted at the two monasteries in a quiet, dark room suitable for meditation and recording EEG. Participants were tested one at a time.

Analyses were performed by means of Brain Vision Analyzer 2.2 (Brain Products GmbH, Germany) and by custom software developed in Matlab R2013b (The MathWorks Inc.). EEG epochs with noise or non-physiological artefacts were removed after visual inspection. Bad channels were interpolated according to Hjorth^53^. For control of ocular artefacts, vertical and horizontal electro-oculogram (EOG) was also recorded and all EEG traces were EOG corrected by means of independent component analysis (ICA^54^).

To improve spatial resolution and reduce the influence of volume conduction between electrodes, current source density (CSD) was additionally applied with the parameters: order of splines = 10, maximal degree of Legendre polynomials = 4, lambda = 1E−7. Because of missing all neighbour electrodes, edge electrodes were excluded from all analyses, so that the number of channels was finally reduced to 50. All analyses were carried out with CSD transformed data from 50 electrodes^12^.

#### Transformation of EEG signals to frequency domain

As in^12^, EEG recordings from all conditions were segmented in equal-sized epochs of 4.096 s duration with 1.024 s overlap. The average number of epochs for each condition/participant was 70 (± 20). A Hanning window with a duration of 20% from the total epoch length was applied to all epochs. This was performed by multiplying the sampled signal values (EEG) by the Hanning function. In such a way, the ends of the time record are forced smoothly to zero regardless of the signal at those parts. This approach is useful when performing spectral analysis on segmented data and avoids artefacts due to abrupt change in the amplitude of the signal at both ends of the segment. After that the fast Fourier transform was computed, yielding the representation of complex values (real and imaginary parts) with a frequency resolution of 0.244 Hz (1/4.096 s).

#### Imaginary part of coherence

Coherence function is a measure of the linear relationship between two signals at a specific frequency (e.g.^55^). In practice, the magnitude of the coherency function is generally used as a measure of coherence. Coherence function *C*_*xy*_*(f)* is defined as the normalized cross-spectrum (the cross-correlation between two time series in the frequency domain):

$${C}_{xy}\left(f\right)=\frac{\sum Sxy\left(f\right)}{\sqrt{\sum Sxx(f)\sum Syy(f)}},$$

where *Sxy* is the cross-spectral density (cross-spectral energy distribution per unit time) between two signals, and *Sxx* and *Syy* are the autospectral densities (energy distribution per unit time computed for one time series) for signals *x* and *y*, respectively. Coherence is defined as the magnitude of coherence function:

$${Coh}_{xy}\left(f\right)=\left|{C}_{xy}\left(f\right)\right|=\left|\frac{\sum Sxy\left(f\right)}{\sqrt{\sum Sxx(f)\sum Syy(f)}}\right|.$$

The estimated coherence for a given frequency ranges between 0 and 1, with a value of 0 indicating that the two signals are perfectly uncorrelated, and a value of 1 indicating the perfect correlation.

Instead of estimating the magnitude of the coherence function, Nolte et al.^52^ proposed using only the imaginary part of the coherency to investigate directed brain interactions. This is because the imaginary part of the coherency excludes coherent sources with zero phase lag and therefore reduces the effect of field spread due to volume conduction. The imaginary part of coherency (*ICoh*) is defined as

$${ICoh}_{xy}\left(f\right)=Im\left({C}_{xy}\left(f\right)\right)=Im\left(\frac{\sum Sxy\left(f\right)}{\sqrt{\sum Sxx(f)\sum Syy(f)}}\right) ,$$

where *Im* denotes the imaginary part. The imaginary part of coherency for a given frequency varies between − 1 and 1. If the value is positive, signals *x* and *y* are interacting and *y* is earlier than *x*, indicating that information is flowing from *y* to *x*. For a similar approach, see Yordanova et al.^12^.

#### Connectivity analysis of EEG signals

The imaginary part of coherency is only sensitive to functional coupling between two signals which are time-lagged to each other. Therefore, as far as volume conduction does not cause a time-lag, this measure is less sensitive to common sources effects^52^. *ICoh* was obtained for all electrode combinations for each condition and each participant. In this study, we investigated the absolute value of *ICoh*. Additionally, before submitting it to statistical analysis, *ICoh* was three-point smoothed and, in order to normalize distribution, Fisher z-transformation was applied. Fisher z-transformation is given by the equation:

$$z\, = \,1/2 \, \ln \left( {\left( {1\, + \,r} \right)/\left( {1 - r} \right)} \right)\, = \,\arctan h\left( r \right),$$

where *ln* is the natural logarithm, *arctanh* is the inverse hyperbolic tangent function, and *r* is the actual value. Measurable parameters were *ICoh* values for frequency ranges theta (4.1–7.1 Hz), slow alpha (8.8–12 Hz), fast alpha (12.2–14.9 Hz), and beta (15–20 Hz)^12^.

### Statistical analysis of FP and MF networks

The statistical evaluation of FP and MF networks were performed for selected electrode pairs using analysis of variance with repeated measures (ANOVA), analysis of covariance with *Mediation Expertise* as a covariate (ANCOVA), correlation and linear and quadratic regression analyses, as detailed in the results. The difference values for all variables included in the ANOVAs and ANCOVAs were tested for normality of distribution using Kolmogorov–Smirnov and Shapiro–Wilk tests. For none of the variables was *p* < 0.05 indicating normality of distribution (Shapiro–Wilk test, *Statistic* > 0.9, *df* = 22).

## Results

### Effects of meditation states on fronto-parietal networks

**Figure 1:**
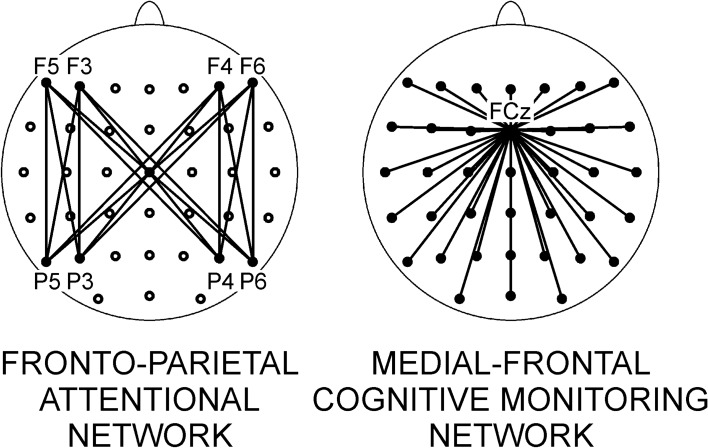
Schematic presentation of attentional and cognitive monitoring networks: fronto-parietal (left) and medial frontal (right). Respective electrodes are labelled according to the International 10–20 system. Electrodes used for analyses are presented in black.

To assess the effects of meditation on FP networks, specific frontal-parietal electrode pairs were selected for analysis. As depicted in Fig. 1, the pairs included four frontal (F5, F3, F4, F6) and four parietal (P5, P3, P4, P6) left- and right-hemisphere electrodes. The resulting 16 frontal-parietal pairs captured the connections of each frontal electrode with each of four parietal ones and vice versa. These electrodes were chosen to cover a possible distinction between dorsal and ventral attention networks^28,42,56^. To assess specific effects of meditation states on FP connectivity, difference *ICoh* values were computed by subtracting *ICoh* during rest from *ICoh* in a given meditation condition in a pair-wise manner. Hence, positive values reflect meditation-related increases. Difference *ICoh* values were subjected to a 3 × 4 × 4 repeated-measures analysis of variance with within-subjects factors *Meditation Condition* (FAM vs. OMM vs. LKM), *Frontal Nodes* (4 levels corresponding to 4 frontal electrodes) and *Parietal Nodes* (4 levels corresponding to 4 parietal electrodes). This analysis design was chosen to assess the possible contribution of specific nodes in the fronto-parietal networks, which would produce main *Frontal* or *Parietal* effects, in addition to the contribution of single pairs, which would be reflected by *Frontal* × *Parietal* interactions. In a second analysis-step, we included *Meditation Expertise* (in hours) as a covariate to test if expertise influenced the involvement of FP networks during specific meditation states. Analyses were performed for theta, slow alpha, fast alpha, and beta frequency bands following previous research according to which FP networks may operate in these frequency ranges^40–42^.

**Figure 2:**
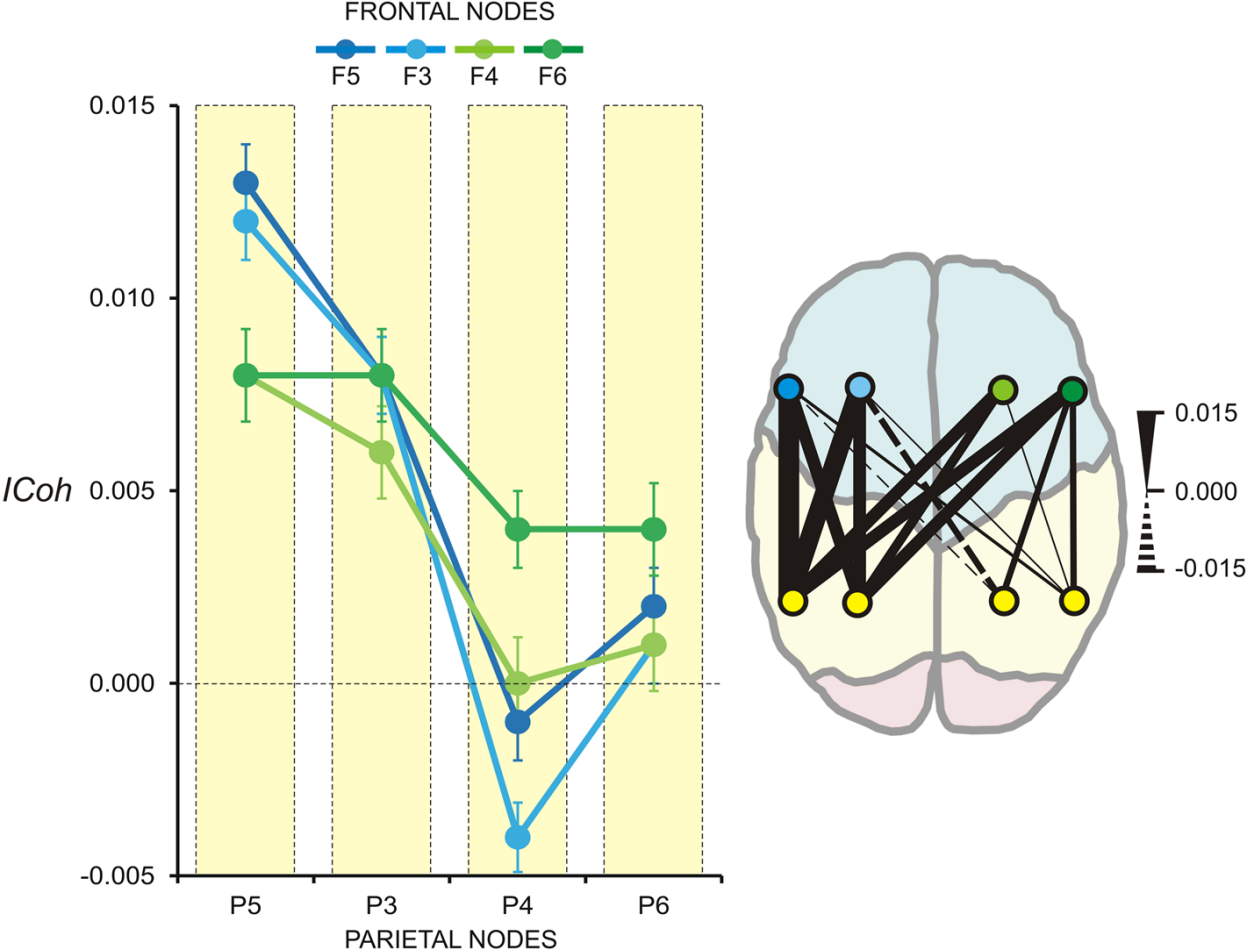
Connectivity of fronto-parietal theta networks during meditation. Left: Group mean ± *SE* of *ICoh* difference (meditation minus rest) in the theta frequency band (4–7 Hz); FAM, OMM and LKM pooled together. *ICoh* difference for each fronto-parietal electrode pair is presented, e.g., P5-F5, P5-F3, P5-F4, P5-F6, etc. Parietal nodes P5, P3, P4 and P6 are marked in yellow—X-axis, whereas the frontal nodes are in different colours as indicated in the figure (F5, F3, F4, F6). Y-axis—non-dimensional *ICoh* difference values. Right: Graphical presentation of same results. Thickness of connections codes the value of *ICoh* difference, as indicated. Colours of nodes correspond to the colours used to denote parietal and frontal electrodes in the left panel. The meditation-related intra-hemispheric increase in theta-*ICoh* in the left hemisphere, common to FAM, OMM and LKM, is demonstrated.

As depicted in Fig. 2, in the theta range FP networks manifested a left-hemisphere increase in connectivity guided by left parietal nodes (*Parietal Node*, *F*(3/63) = 4.9, *p* = 0.03, ${\eta }_{p}^{2}=$ 0.19). This effect did not depend on the type of meditation (no interaction with *Meditation Condition*, *F*(6/126) = 1.1, *p* > 0.3). Adding *Meditation Expertise* as a covariate did not yield any significant interactions.

**Figure 3:**
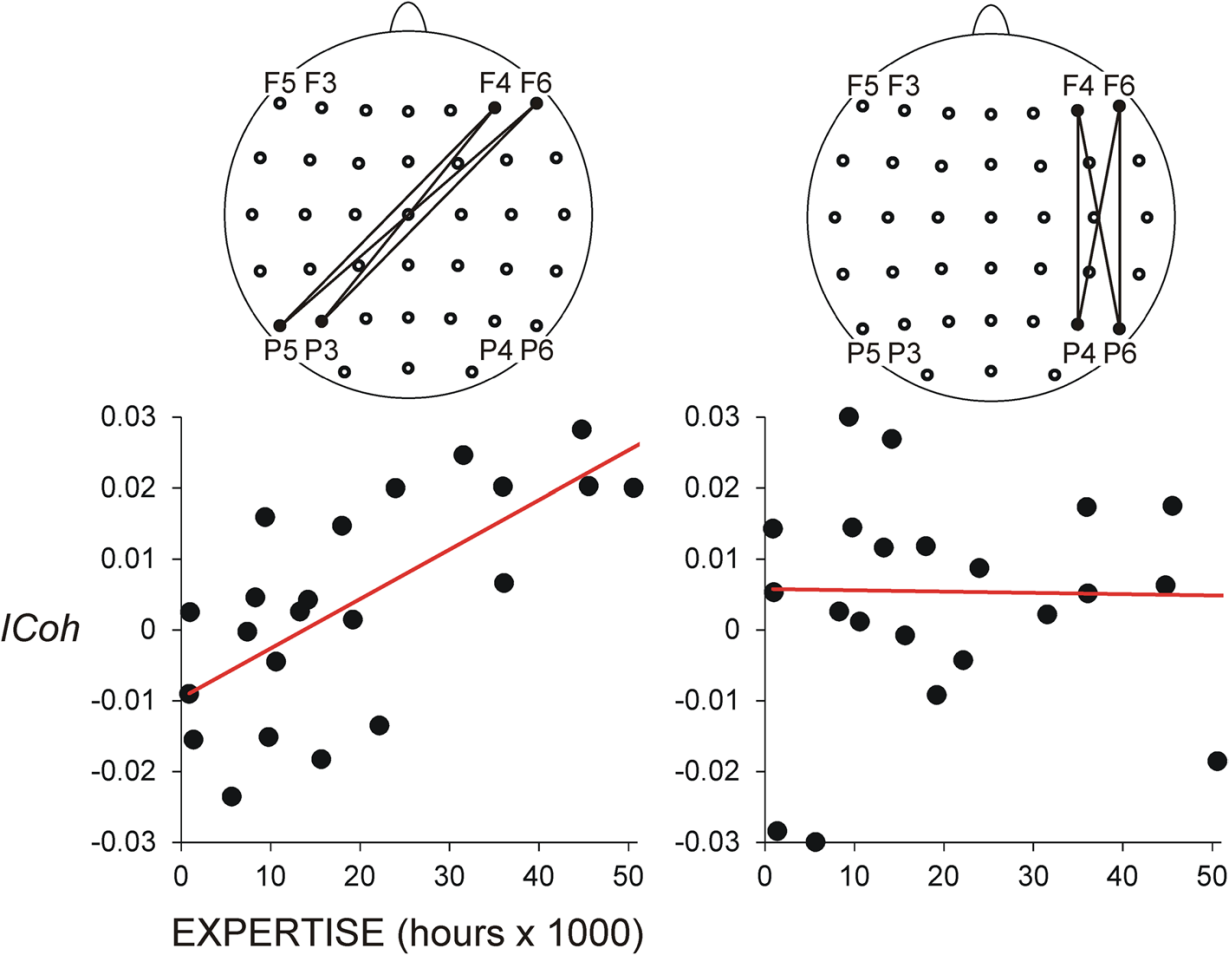
Correlation between fast alpha (12–15 Hz) *ICoh* and meditation expertise. Individual difference *ICoh* values are presented (meditation minus rest); FAM, OMM and LKM pooled together for each individual difference *ICoh* value. Y-axis—non-dimensional *ICoh* difference values. The meditation-related inter-hemispheric linear increase of fast alpha *ICoh* as a function of meditation expertise is depicted on the left for right frontal (F4, F6)—left parietal (P3, P5) electrode pairs. The lack of correlation between meditation-related intra-hemispheric modulation of fast alpha *ICoh* as a function of meditation expertise is shown on the right.

No significant results emerged for slow alpha FP networks, nor were there any interactions with *Meditation Expertise*. Fast alpha FP networks were not modified by *Meditation Condition*, but depended on *Meditation Expertise,* as revealed by a significant ANCOVA *Frontal Nodes* × *Parietal Nodes* × *Meditation Expertise* interaction (*F*(9/180) = 3.5, *p* = 0.03, ${\upeta }_{\mathrm{p}}^{2}=$ 1.15). Specifically, for all meditation conditions, fast alpha synchronization increased with increasing expertise for the connections linking frontal and parietal electrodes of opposite hemispheres. Multivariate ANCOVA (MANCOVA) used to test the effect of *Meditation Expertise* for each single frontal-parietal pair verified that hours of practice predicted the increased fast alpha *ICoh* only for inter-hemispheric FP connections. This effect was most prominent for pairs linking right frontal (F4, F6) with left parietal (P5, P3) nodes (*Meditation Expertise*, *F*(1/20) > 4.5, *p* < 0.05, ${\upeta }_{\mathrm{p}}^{2}$ > 0.19)—Fig. 3 (left). Accordingly, only for these pairs, Pearson correlation coefficients were *r* = − 0.4 ÷ − 0.5, *p* < 0.01. Importantly, intra-hemispheric frontal-parietal connections were not modulated by expertise (*p* > 0.2)—Fig. 3 (right).

Likewise, no main or interaction effects of *Meditation Condition* were found for FP networks in the beta range. The significant *Frontal Node* × *Parietal Node* × *Meditation Expertise* interaction yielded by ANCOVA (*F*(9/180) = 3.5, *p* = 0.05, ${\upeta }_{\mathrm{p}}^{2}=$ 0.17), reflected a practice-dependent increase in right frontal—left parietal connections in the beta range, similar to those found in the fast alpha range (MANCOVA, *F*(1/20) > 4.3, *p* < 0.05, ${\upeta }_{\mathrm{p}}^{2}$ > 0.18; Pearson correlation coefficients, *r* = − 0.4 ÷ − 0.5, p < 0.05).

These results indicate that the specific types of meditation did not induce distinctive involvement of FP networks: Intra-hemispheric FP theta connectivity was enhanced in the left hemisphere in all three types of meditation (FAM, OMM, and LKM) relative to REST. A second common feature of all three meditation types was that inter-hemispheric frontal-parietal connections in fast alpha and beta bands linking mostly right-frontal regions with left-parietal regions manifested increased connectivity as a function of meditation expertise.

### Effects of meditation on the medial frontal network

As shown in Fig. 1, the cognitive monitoring (MF) network was assessed using *ICoh* measures of all pairs of electrodes guided by the FCz electrode. It has been demonstrated that the midfronto-central region represents a fulcrum capturing and controlling activity from the dorsal ACC and medial frontal lobe responsible for performance monitoring and re-adjustment^48^, where the connections with dorsolateral frontal and parietal regions are of specific relevance^47^. As in the previous analysis, difference *ICoh* values were used. They were obtained by subtracting measures during REST from measures during each of the three meditation states, separately for theta, slow alpha, fast alpha, and beta frequency bands. In the present analysis, five frontal (F5, F3, Fz, F4, F6), central (C5, C3, Cz, C4, C6), centro-parietal (CP5, CP3, CPz, CP4, CP6), parietal (P5, P3, Pz, P4, P6), parieto-occipital (P7, PO3, POz, PO4, P8) and occipital (PO7, O1, Oz, O2, PO8) electrodes were used to form the *Region* (6 levels) and *Laterality* (5 levels) within-subjects variables of a repeated measures ANOVA. A third within-subjects factor was *Meditation Condition* with three levels (FAM, OMM, LKM). As a second step, ANCOVA was run by including *Meditation Expertise* as a covariate.

No significant main or interaction effects were found for slow and fast alpha frequency bands. As detailed below, MF networks in the theta and beta frequency ranges were involved in sustaining specific meditation states.

**Figure 4:**
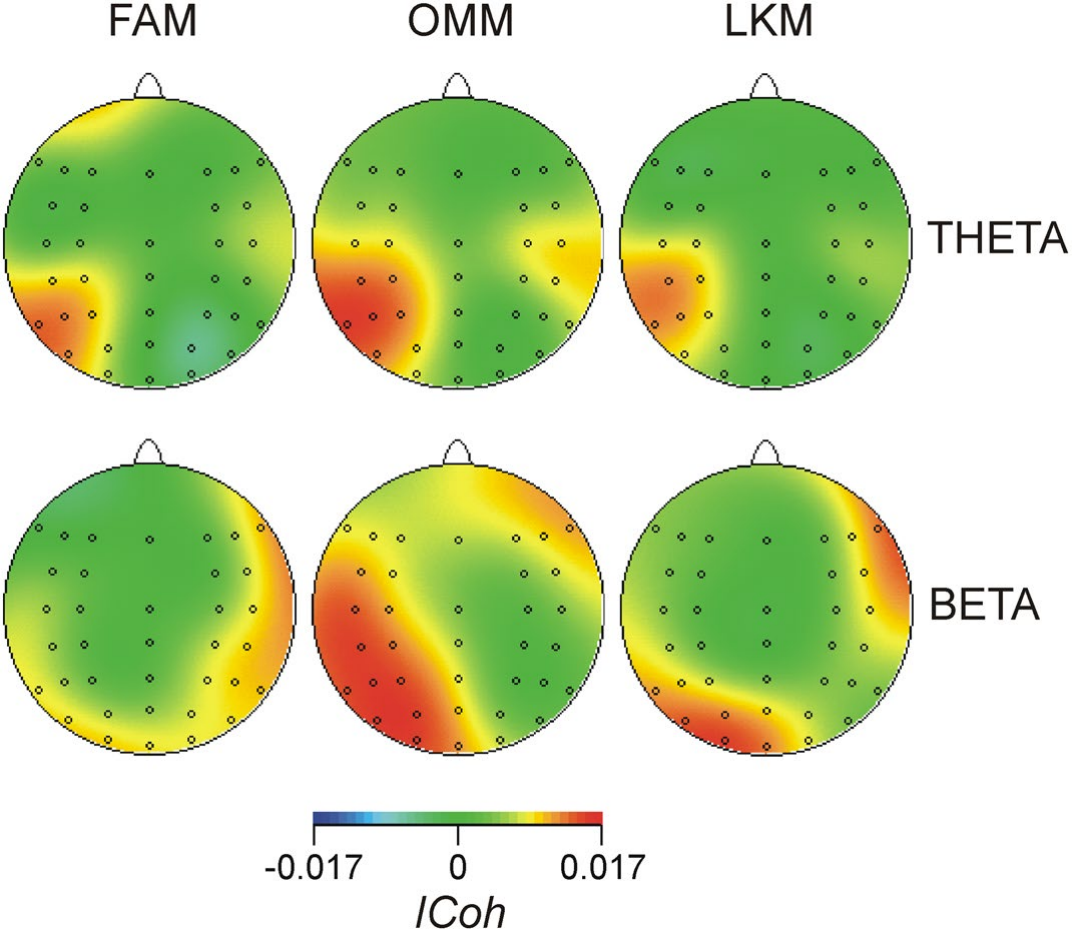
Connectivity of the medial frontal network during meditation. Topography maps of grand average *ICoh* difference are presented (FAM, OMM and LKM minus REST). Upper row presents theta frequency range (4–7 Hz), and lower row—beta frequency range (15–20 Hz). Red colour indicates meditation-related increase.

**Figure 5:**
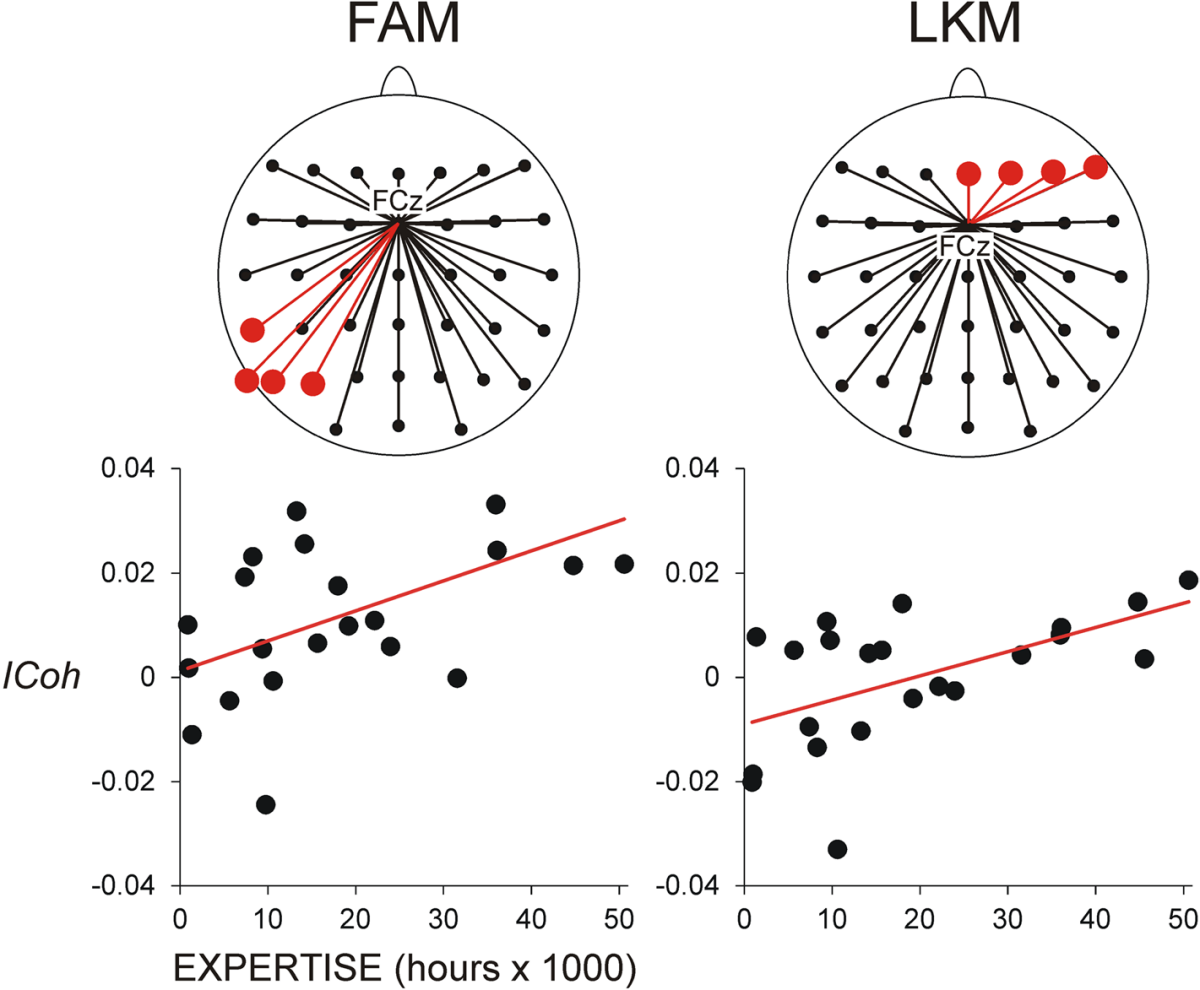
Linear associations between theta (4–7 Hz) *ICoh* of the medial frontal network (presented as individual difference values, meditation minus rest) and meditation expertise. Upper panel: Graphical maps indicating relevant electrodes where the correlations between theta *ICoh* and meditation expertise were significant (red circles and connections). Lower panel: Linear increase of theta *ICoh* as a function of meditation expertise. Positive *ICoh* difference values reflect meditation-related increase. The linear increase of theta *ICoh* for FCz-guided left parieto-occipital pairs as a function of meditation expertise is demonstrated for FAM (left); the linear increase of theta *ICoh* for FCz-guided right frontal pairs as a function of meditation expertise is demonstrated for LKM (right). Y-axis presents non-dimensional *ICoh* difference values.

Figure 4 demonstrates that during all meditation conditions, MF theta networks manifested increased synchronization at posterior regions (centro-parietal, parietal and parieto-occipital) of the left hemisphere (*Laterality*, *F*(4/84) = 5.9, *p* = 0.005, ${\upeta }_{\mathrm{p}}^{2}=$ 2.2; *Region* × *Laterality*, *F*(20/420) = 2.98, *p* < 0.02, ${\upeta }_{\mathrm{p}}^{2}=$ 0.12). Adding *Meditation Expertise* as a covariate revealed that *Meditation Expertise* predicted theta synchronization in specific regions depending on the meditation condition (*Meditation Condition* × *Region* × *Laterality* × *Meditation Expertise*, *F*(40/800) = 2.1, *p* = 0.04, ${\upeta }_{\mathrm{p}}^{2}=$ 0.1). MANCOVA tests of the effect of *Meditation Expertise* for single electrode pairs in each meditation condition demonstrated that during FAM, theta synchronization between FCz and left parietal regions (P7, P5, P3, CP5) increased as a function of expertise (*F*(1/20) > 4.4, *p* < 0.05, ${\upeta }_{\mathrm{p}}^{2}$ > 0.18), whereas during LKM, *Meditation Expertise* co-varied with the increased theta synchronization between FCz and right frontal regions F6, F4, Fz (*F*(1/20) > 8.5, *p* < 0.008, ${\upeta }_{\mathrm{p}}^{2}$ > 0.29). These relationships are shown in Fig. 5.

Figure 4 further demonstrates that in the beta range FAM was associated with increased synchronization of MF connections in the right hemisphere, whereas OMM was associated with increased synchronization in the left posterior hemisphere (*Meditation Condition* × *Laterality*, *F*(8/168) = 3.4, *p* = 0.03, ${\upeta }_{\mathrm{p}}^{2}=$ 0.14). Accordingly, there was a significant difference between FAM and OMM over the left hemisphere (*F*(1/21) = 6.4, *p* = 0.02, ${\upeta }_{\mathrm{p}}^{2}=$ 0.23), and at posterior regions of the right hemisphere (*F*(1/21) = 3.7, *p* = 0.05, ${\upeta }_{\mathrm{p}}^{2} =$ 0.18). During LKM, left posterior and right anterior increases in synchronization were observed (*Meditation Condition* × *Region* × *Laterality*, *F*(40/840) = 2.2, *p* = 0.04, ${\upeta }_{\mathrm{p}}^{2}=$ 0.1; *Region* × *Laterality* in LKM, *F*(20/420) = 2.5, *p* = 0.05, ${\upeta }_{\mathrm{p}}^{2}=$ 0.11). However, no significant main or interaction effects of the covariate *Meditation Expertise* were found.

### Regression analyses

To further explore the effects of the *Meditation Expertise* covariate in light of previous results^57^, measures of FP and MF networks were included in linear and quadratic regression models to test for the existence of linear or non-linear, U-shaped dependencies between meditation-specific changes in connectivity and *Meditation Expertise*. Confirming results of MANCOVAs, the analyses yielded significant solutions of linear regression models for inter-hemispheric FP synchronization in fast alpha and beta bands (*F*(1/21) = 3.8 ÷ 6.8, p = 0.05 ÷ 0.01). Specifically, increasing practice duration was associated with increased synchronization in the fast alpha and beta ranges between frontal and parietal regions of opposite hemispheres (Fig. 3). Likewise, for MF networks, only synchronization in the theta frequency range was linearly associated with *Meditation Expertise* such that long-duration practice predicted increased theta synchronization between the medial fronto-central region and the left parietal hemisphere during FAM, and the right frontal regions during LKM (*F*(1/21) = 4.5 ÷ 8.5, *p* = 0.05 ÷ 0.008)—Fig. 5.

**Figure 6:**
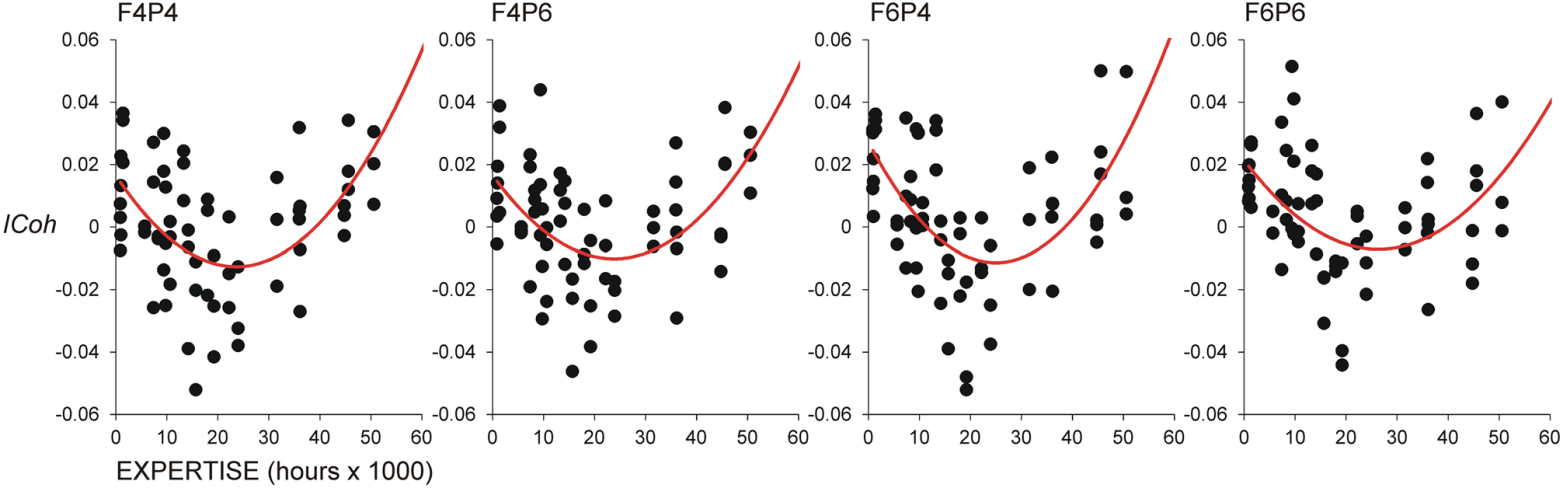
Regression analyses for intra-hemispheric connections in the right hemisphere for theta frequency range vs. meditation expertise. Individual difference *ICoh* values are included for FAM, OMM and LKM (meditation minus rest). Positive *ICoh* difference values reflect meditation-related increase. Right hemisphere pairs are indicated above each graph. Y-axis—non-dimensional *ICoh* difference values. U-shape dependency on meditation expertise indicating a most pronounced increase in synchronization in least and most experienced meditators is demonstrated.

Notably, only for FP theta synchronization, there were significant solutions in the quadratic models. As illustrated in Fig. 6, intra-hemispheric connections in the right hemisphere manifested a U-shape dependency on *Meditation Expertise* indicating more pronounced increase in synchronization in least and most experienced meditators (*F*(2/19) = 3.5 ÷ 10.9, *p* = 0.05 ÷ 0.001). These quadratic models proved significant for all meditation states, pointing to a generalized effect of *Meditation Expertise* on changes of FP theta synchronization, irrespective of the type of meditation.

**Figure 7:**
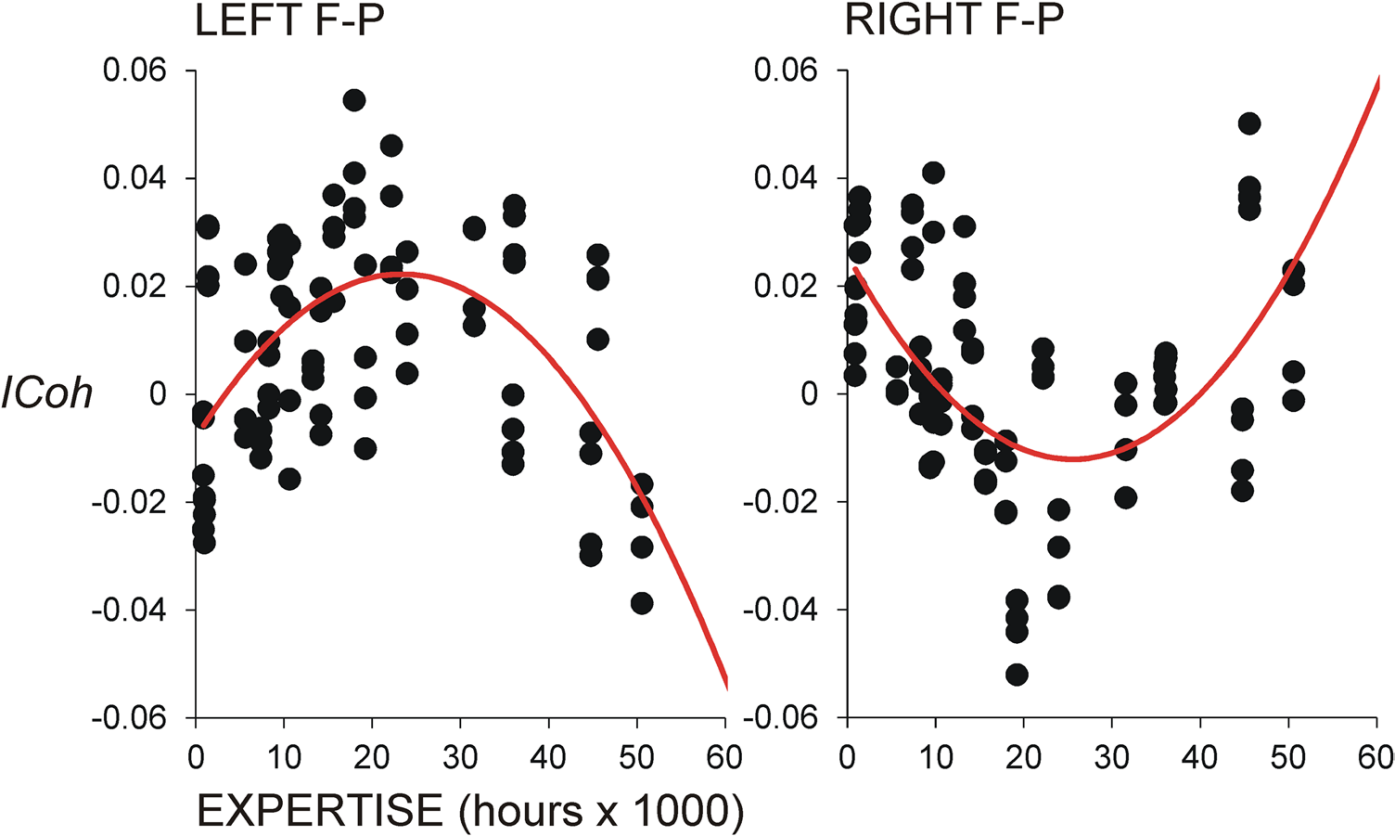
Results from all left and right within-hemisphere fronto-parietal (F-P) pairs for LKM. Clear opposite tendencies in the two hemispheres compared to Fig. 6 are illustrated. Designations are the same as in Fig. 6.

In contrast, as depicted in Fig. 7, significant inverted U-shape solutions were yielded for the left-hemisphere intra-hemispheric connections during LKM indicating a least pronounced synchronization for least and most experienced meditators (*F*(2/19) = 3.5 ÷ 6.5, *p* = 0.05 ÷ 0.007). These inverted U-shape associations between intra-hemispheric FP connections in the left hemisphere and *Meditation Expertis*e were also found during FAM and OMM but they did not reach significance.

### Control analyses

As control analyses, *ICoh* values were computed for 11 individuals (mean age = 45.5 ± 12.6 years, 9 females) with less than 250 h of meditation experience in secular mindfulness or Buddhist traditions. These short-term meditators further practiced the instructions of FAM, OMM and LKM for 10 days before the study, 20 min per day for each form of meditation. EEG records and analyses were conducted following the same design and procedures used for data acquisition and analysis in experienced meditators. The only significant result that was yielded was an increased involvement of frontal nodes of slow alpha FP network in the right hemisphere (*Frontal Node*, *F*(3/30) = 4.8, *p* = 0.03), which was similar across three meditation states (*Meditation Condition* × *Frontal Node*, *F*(6/60) = 0.1, *p* > 0.9).

In another control analysis, we tested if the common involvement of left posterior theta FP and MF networks may reflect a generalized network activity induced by the integrated meditation training of participants or carry over effects from one meditation state to another in the current experimental design. To check for the existence of such a generalized network we performed analyses, in which we used the original (un-subtracted) *ICoh* values in the theta band. It was hypothesized that a common connectivity pattern would be shared across all three meditation styles. Although non-significant, the observations provided evidence that despite the integrated training of participants, and despite the integrated experimental session including the three meditation styles, the connectivity patterns of FP and MF networks were distinct across meditation states (see Supplementary Information). Therefore, a generalized network may not be responsible for the specific left-lateralized increase in FP and MF connectivity common for all states of mediation. Rather, an enhanced involvement of left-lateralized nodes, irrespective of style-specific network properties, appears as a common activation induced by any type of meditation.

## Discussion

Executive top-down processes guiding voluntary allocation of attention are associated with the fronto-parietal brain networks^26,29,58–60^, whereas executive processes of cognitive monitoring are mainly linked to the medial frontal networks^27,44,45,49^. The present study characterized the functional connectivity of these FP and MF networks in major forms of meditation (focused attention, open monitoring and loving kindness meditation) in long-term practitioners, as contrasted with a non-meditative resting state. The main objective was to explore the role of executive brain processes in sustaining different forms of meditation as a function of meditation expertise^6,15,22^. Toward this end, we analysed functional connectivity patterns of FP and MF networks in terms of coherent theta, slow and fast alpha, and beta oscillations, and explored their correlation with meditation expertise.

The results demonstrate that, compared to unexperienced practitioners, meditation states in long-term practitioners induced highly specific connectivity patterns of fronto-parietal and medial frontal networks relative to rest. This observation generally indicates that the executive processes of attentional control and cognitive monitoring have a specific role in supporting brain states of meditation.

### Common and distinct patterns of FP and MF networks in FAM, OMM and LKM

The first major finding of this study is that the synchronization of *fronto-parietal networks* did not differentiate FAM, OMM, and LKM conditions in any of the analyzed frequency bands. Yet, FP networks in long-term meditators manifested an identifiable pattern of organization appearing as a common activation feature of all three types of meditation, which did not exist in novice meditators. In particular, the left parietal nodes of the FP *theta* networks exhibited increased synchronization with frontal regions in the same (left) hemisphere. *Medial frontal* networks in long-term practitioners manifested a similar common *theta* synchronization pattern. These findings demonstrate that the state of meditation in meditation experts engages common executive attention and cognitive monitoring mechanisms supported by synchronized theta networks in the left hemisphere. Meditation style was only distinguished by cognitive monitoring mechanisms associated with lateralized synchronization of beta networks.

We specifically targeted FP and MF networks by focusing on established nodes of these networks^26,29,42,43,48,61,62^. The observed patterns of enhanced theta connectivity of the left parietal region, which was common to FP and MF networks, and the distinctive lateralized beta pattern, which differentiated the MF network in FAM and OMM, resemble the more general picture of connectivity extracted in an initial analysis of the same data set^12^. In that study, a wide range of connections (not limited to FP and MF electrode pairs), was used. Similar to the present results, the functional connectivity of multiple theta connections was increased in the left hemisphere in all forms of meditation, and lateralized beta connectivity differentiated the FAM and OMM styles.

The current targeted analysis of FP and MF networks indicates that the previously identified unique centre of increased theta connectivity in the left posterior regions is associated with the executive attentional and cognitive monitoring systems when experts meditate. In support, *theta* has previously been identified as a major operating rhythm of cognitive control^47^, which supports the controlled signalling from prefrontal to medial frontal regions^63^, the cognitive monitoring and coordinating functions of the MF system^43,48,49,62,64^, and the guided voluntary allocation of focused attention mediated by FP networks^40,42,60^. The common engagement of theta frequency is also consistent with previously found shared involvement of medial frontal structures (pre/supplementary motor cortices, dorsal anterior cingulate cortex) and fronto-polar cortex in different meditation styles including FAM, OMM and LKM^20^.

Localizing the theta synchronization hub in *parietal regions* is in line with existing neurocognitive models regarding the role of these regions. These models propose that the core function of the lateral parietal activity either is to support bottom-up attentional capture^65,66^, or to mediate a “cross-modal integrative hub” combining bottom-up multimodal inputs with top-down controlling signals^67,68^. More specifically, this multi-modal hub is assumed to integrate information in order to enhance awareness (comprehension and meaning), manipulate mental events, and reorient attention to relevant information^68^. In a similar manner, the lateral parietal area has also been implicated in the formation of attentional “priority maps” which represent environmental bottom-up features selected by the top-down focus of attention and are dynamically shaped by task-specific motor, cognitive and motivational variables^69^. The present results suggest that in experienced meditators such an integrative cognitive control hub in the *left hemisphere* is enhanced during all three meditation types (Figs. 4 and 5). This suggestion is in line with (1) fMRI and MEG findings of a prominent cluster of left-hemispheric activation across different meditation types^16,70,71^, (2) evidences about more expressed structural differences in the left hemisphere of long-term meditation practitioners than in novices^72–74^, (3) current theoretical developments emphasizing on the leading role of the left hemisphere in executive top-down regulation of meditation states^22^.

The currently found hemispheric asymmetry can be understood within several perspectives. One account is a possible lateralized involvement of dorsal and ventral attention networks^26^, whereby the dorsal network controlling top-down focusing, orienting and sustaining attention is bi-lateral, and the ventral network controlling attention re-orienting to distracting but behaviourally relevant or salient events is right-lateralized^26,28,29,75^. Since the ventral network has been found to be suppressed during top-down guided focused attention to protect goal-driven behaviour^56,76,77^, the observed here left-hemisphere dominance might emerge. Second, Posner and Rothbart^27^ posit that executive influences on awareness involve differentially the two hemispheres: The right hemisphere may be engaged in top-down controlled sensory awareness, whereas the left hemisphere may be responsible for directing motor attention in relation to voluntary initiation and control of behaviour^78–81^. Although the leftward lateralization during meditation may have a complex origin, the present results demonstrate its dominant role in top-down executive functions during all three forms of meditation in long-term practitioners.

Regarding the neural coupling in the beta range, medial-frontal connections were more strongly synchronized in the left hemisphere for OMM and in the right hemisphere for FAM. This condition-dependent lateralization of beta synchronization corresponds to the broader pattern reported in our previous analysis of the data^12^. However, the current analysis qualifies this, in that the lateralized beta connectivity was only observed for the MF cognitive monitoring network and not for the attentional FP networks. This observation helps to refine the functional involvement of beta oscillations during meditation. It implies that the lateralized beta synchronization may be associated with a top-down amplification of attended information^82^, or with mediating the link between top-down attentional selection and awareness^42,83–86^. Also, since medial frontal beta activity has been associated with monitoring of conflicts and subsequent behavioural adjustments and adaptation^64^, the right- vs. left lateralization of MF beta connectivity in FAM vs. OMM may reflect different types of conflict monitoring, distracter inhibition and adaptation: sensory during FAM and behavioural during OMM^78,80^.

### Modulation of FP and MF networks by meditation expertise: common and distinct patterns of modulation

The present results demonstrate that connectivity of FP and MF networks depend on the extent of meditation expertise. Importantly, this expertise-dependent modulation was different for fronto-parietal and medial frontal networks, suggesting an overall difference between neuroplastic dynamics of these executive networks in the course of meditation practice.

Fronto-parietal modulations by expertise were observed for all three meditation types, suggesting a generalized effect of meditation training on FP functions. Also, the effects of expertise on FP networks were different according to network frequency. Specifically, *intra-hemispheric* FP *theta* connections depended *non-linearly* on expertise following a U-shape model. In contrast, *inter-hemispheric* FP connections in *fast alpha and beta bands* manifested a *linear* increase in connectivity as a function of expertise.

Since the expertise-dependent U-shaped trajectory of FP theta connectivity was present in all three meditation states (FAM, OMM, LKM), it seems unlikely that practice-related neuroplasticity reflects changes in the control of attentional focus (narrow vs. wide), or in the amount or specificity of the content of meditation (e.g., object-specific, objectless, emotional/mental state). Rather, with regard to the previously reported domain-unspecific effects of task difficulty on the activity in lateral parietal sub-regions^67^, non-linear modulations by expertise may reflect training-related changes in mental/neurocomputational *effort*. From this cognitive effort perspective, an initial phase of increased mobilization of neuronal resources in response to training, wouldbe followed by a more efficient use of (less) neuronal resources^57,87^. This predictethrough continued practice—be followed by a more efficient use of (less) neuronal resources^57,87^. This predicted non-linear dependency of FP theta connectivity was only observed in the left hemisphere, appearing stable in LKM (Fig. 7), possibly in relation to the added load of emotional/motivational processing in this condition^88,89^.

A further intriguing observation is the opposing pattern of expertise-dependent FP theta synchronization in the two hemispheres. These co-existing opposite effects suggest that top-down attentional regulation of effort is under dynamic inter-hemispheric control in terms of a counter-balanced involvement of the left- and the right-hemisphere FP networks. Long-term meditation training may therefore disentangle the two hemispheres leading to alternating dominance of either the left or the right hemisphere (i.e., a dominating role of left-lateralized FP networks in least and most experienced meditators, with a reverse pattern—a dominating role of right-lateralized FP networks—in intermediate-level meditators). In other words, neuroplasticity due to meditation training may be linked to dynamic shifts of attentional effort control across hemispheres. The results further suggest that attentional effort control can be guided predominantly by either the left or the right hemisphere depending on the level of attentional training and/or simultaneously activated processes^26,28,29,39^. Importantly, these non-linear modulations of theta FP networks by expertise coexisted with a progressive increase of inter-hemispheric fast-frequency FP connections. From a neuro-functional perspective, this result may reflect facilitation of conscious access^42,82,84–86^ and a transference of attention-dependent conscious access to higher-frequency networks, as suggested previously^17,25^. It may be further assumed that these differential frequency-dependent effects of expertise capture specific meditation-related neuroplasticity of FP networks associated with attentional control of effort and conscious access.

In parallel, effects of expertise were found for medial frontal theta networks. In contrast to FP networks, these expertise-dependent modulations were linear and also depended on the type of meditation: during FAM, theta synchronization increased as a function of meditation expertise at left parietal regions, whereas during LKM, it increased at right frontal regions, with no specific regions involved in OMM (Fig. 5). Since theta oscillations represent a major signal supporting the maintenance/monitoring of internal representations^48,64,90–92^, these observations imply that meditation training facilitates access to specific contents (mental representations) that require monitoring and adaptation depending on the type of meditation. Indeed, consistent with observations for LKM, functional imaging studies have demonstrated that the *right* prefrontal cortex may be particularly critical for voluntary regulation of emotions, and for suppression of negative emotions in particular, as well as for self-regulation and self-control^93–95^. Also, the right ventromedial cortex has been shown to have a unique role in integrating cognition and affect to produce the empathic response^96^. Likewise, consistent with current results for FAM, attention priority maps of sensorimotor goals appear to be supported by the parietal regions^68,69^. Accordingly, the lack of an intended mental object in OMM corresponds to the lack of any specific region of expertise-dependent increase of MF theta connectivity in this condition. In combination with practice-based modulations of FP networks, these region-specific effects of meditation expertise on MF theta connectivity may be linked to the progressively increasing subjective feeling of “effortless” maintenance of meditation states in experienced meditators.

Future longitudinal studies on long-term practice of FAM, OMM and LKM may provide further insights and sharpen the present findings, also considering the intrinsic limitations of cross-sectional studies and of a self-reported retrospective assessment of lifetime meditation expertise, which however in our study was enhanced by a careful recollection by the monastic participants, performed over several days, as well as by the practice regularities in monastic life.

## Conclusions

In contrast to unexperienced meditators, the maintenance of meditation states in long-term practitioners is associated with specific connectivity patterns of FP and MF networks.

1. First, executive brain processes of both attention regulation and cognitive monitoring during meditation are supported by networks operating in two major frequency bands—theta and beta—consistent with major operating rhythms of conventional executive networks.
2. Theta and beta connectivity patterns of both FP and MF networks manifest a pronounced lateral asymmetry implying that a focused lateralization of executive systems may represent a major functional mechanism which supports the state of meditation in general, as well as specific meditation states.
3. Connectivity patterns of FP networks were common to all three meditation states, indicating a similar involvement of attentional control across meditation conditions in the course of meditation training. Only the connectivity of medial frontal networks differentiated FAM, OMM and LKM conditions indicating a specific functional relevance for cognitive monitoring in sustaining a particular meditation state.
4. Training of executive systems during long-term meditation practice was accompanied by both non-linear and linear modulations of FP and MF connectivity revealing that shifts in lateralization co-existed with facilitated interactions among lateralized patterns of executive functions. Such practice-based neuroplasticity of executive functions may subserve the emergence of unique mental states in meditation in highly experienced meditators.

## Supplementary Information

Supplementary Information.

## References

[1] Van Aalderen JR, Donders AR, Giommi F, Spinhoven P, Barendregt HP, Speckens AE (2012). The efficacy of mindfulness-based cognitive therapy in recurrent depressed patients with and without a current depressive episode: A randomized controlled trial. Psychol. Med..

[2] Davis DM, Hayes JA (2011). What are the benefits of mindfulness? A practice review of psychotherapy-related research. Psychotherapy.

[3] Goyal M, Singh S, Sibinga EM, Gould NF, Rowland-Seymour A, Sharma R (2014). Meditation programs for psychological stress and well-being: A systematic review and meta-analysis. JAMA Intern. Med..

[4] Gu J, Strauss C, Bond R, Cavanagh K (2015). How do mindfulness-based cognitive therapy and mindfulness-based stress reduction improve mental health and wellbeing? A systematic review and meta-analysis of mediation studies. Clin. Psychol. Rev..

[5] Hofmann SG, Grossman P, Hinton DE (2011). Loving-kindness and compassion meditation: Potential for psychological interventions. Clin. Psychol. Rev..

[6] Lutz A, Slagter HA, Dunne JD, Davidson RJ (2008). Attention regulation and monitoring in meditation. Trends Cogn. Sci..

[7] Davidson RJ, Dahl CJ (2018). Outstanding challenges in scientific research on mindfulness and meditation. Perspect. Psychol. Sci..

[8] Dorjee D (2016). Defining contemplative science: The metacognitive self-regulatory capacity of the mind, context of meditation practice and modes of existential awareness. Front. Psychol..

[9] Schöne B, Gruber T, Graetz S, Bernhof M, Malinowski P (2018). Mindful breath awareness meditation facilitates efficiency gains in brain networks: A steady-state visually evoked potentials study. Sci. Rep..

[10] Malinowski P, Shalamanova L (2017). Meditation and cognitive ageing: The role of mindfulness meditation in building cognitive reserve. J. Cogn. Enhan..

[11] Van Dam NT, Van Vugt MK, Vago DR, Schmalzl L, Saron CD, Olendzki A (2018). Mind the hype: A critical evaluation and prescriptive agenda for research on mindfulness and meditation. Persp. Psychol. Sci..

[12] Yordanova J, Kolev V, Mauro F, Nicolardi V, Simione L, Calabrese L (2020). Common and distinct lateralised patterns of neural coupling during focused attention, open monitoring and loving kindness meditation. Sci. Rep..

[13] Hasenkamp W, Wilson-Mendenhall CD, Duncan E, Barsalou LW (2012). Mind wandering and attention during focused meditation: A fine-grained temporal analysis of fluctuating cognitive states. NeuroImage.

[14] Isbel B, Summers MJ (2017). Distinguishing the cognitive processes of mindfulness: Developing a standardised mindfulness technique for use in longitudinal randomised control trials. Conscious. Cogn..

[15] Malinowski P (2013). Neural mechanisms of attentional control in mindfulness meditation. Front. Neurosci..

[16] Manna A, Raffone A, Perrucci MG, Nardo D, Ferretti A, Tartaro A (2010). Neural correlates of focused attention and cognitive monitoring in meditation. Brain Res. Bull..

[17] Fell J, Axmacher N, Haupt S (2010). From alpha to gamma: Electrophysiological correlates of meditation-related states of consciousness. Med. Hypotheses.

[18] Cahn BR, Polich J (2006). Meditation states and traits: EEG, ERP, and neuroimaging studies. Psychol. Bull..

[19] Dahl CJ, Lutz A, Davidson RJ (2015). Reconstructing and deconstructing the self in three families of meditation. Trends Cogn. Sci..

[20] Fox KCR, Dixon ML, Nijeboer S, Girn M, Floman JL, Lifshitz M (2016). Functional neuroanatomy of meditation: A review and meta-analysis of 78 functional neuroimaging investigations. Neurosci. Biobehav. Rev..

[21] Vago DR, Silbersweig DA (2012). Self-awareness, self-regulation, and self-transcendence (S-ART): A framework for understanding the neurobiological mechanisms of mindfulness. Front. Hum. Neurosci..

[22] Raffone A, Marzetti L, Del Gratta C, Perrucci MG, Romani GL, Pizzella V (2019). Toward a brain theory of meditation. Prog. Brain Res..

[23] Smallwood J, McSpadden M, Schooler JW (2007). The lights are on but no one's home: Meta-awareness and the decoupling of attention when the mind wanders. Psychon. Bull. Rev..

[24] Lippelt DP, Hommel B, Colzato LS (2014). Focused attention, open monitoring and loving kindness meditation: Effects on attention, conflict monitoring, and creativity—a review. Front. Psychol..

[25] Lutz A, Greischar LL, Rawlings NB, Ricard M, Davidson RJ (2004). Long-term meditators self-induce high-amplitude gamma synchrony during mental practice. Proc. Natl. Acad. Sci. USA.

[26] Corbetta M, Shulman GL (2002). Control of goal-directed and stimulus-driven attention in the brain. Nat. Rev. Neurosci..

[27] Posner M, Rothbart MK (1998). Attention, self-regulation and consciousness. Philos. Trans. R. Soc. Lond. B.

[28] Chica AB, Paz-Alonso PM, Valero-Cabré A, Bartolomeo P (2013). Neural bases of the interactions between spatial attention and conscious perception. Cereb. Cortex.

[29] Vossel S, Geng JJ, Fink GR (2014). Dorsal and ventral attention systems: Distinct neural circuits but collaborative roles. Neuroscientist.

[30] Dehaene S, Kerszberg M, Changeux J-P (1998). A neuronal model of a global workspace in effortful cognitive tasks. Proc. Natl. Acad. Sci. USA.

[31] Dehaene S, Changeux JP (2011). Experimental and theoretical approaches to conscious processing. Neuron.

[32] Rees G (2013). Neural correlates of consciousness. Ann. NY Acad. Sci..

[33] Bressler SL, Menon V (2010). Large-scale brain networks in cognition: Emerging methods and principles. Trends Cogn. Sci..

[34] Egner T, Monti JM, Trittschuh EH, Wieneke CA, Hirsch J, Mesulam MM (2008). Neural integration of top-down spatial and feature-based information in visual search. J. Neurosci..

[35] Menon V (2013). Developmental pathways to functional brain networks: Emerging principles. Trends Cogn. Sci..

[36] Rottschy C, Langner R, Dogan I, Reetz K, Laird AR, Schulz JB (2012). Modelling neural correlates of working memory: A coordinate-based meta-analysis. NeuroImage.

[37] Rottschy C, Caspers S, Roski C, Reetz K, Dogan I, Schulz JB (2013). Differentiated parietal connectivity of frontal regions for "what" and "where" memory. Brain Struct. Funct..

[38] Hardwick RM, Rottschy C, Miall RC, Eickhoff SB (2013). A quantitative meta-analysis and review of motor learning in the human brain. NeuroImage.

[39] Corbetta M, Shulman GL (2011). Spatial neglect and attention networks. Annu. Rev. Neurosci..

[40] Daitch AL, Sharma M, Roland JL, Astafiev SV, Bundy DT, Gaona CM (2013). Frequency-specific mechanism links human brain networks for spatial attention. Proc. Natl. Acad. Sci. USA.

[41] Sadaghiani S, Kleinschmidt A (2016). Brain networks and α-oscillations: Structural and functional foundations of cognitive control. Trends Cogn. Sci..

[42] Yordanova J, Kolev V, Verleger R, Heide W, Grumbt M, Schürmann M (2017). Synchronization of fronto-parietal beta and theta networks as a signature of visual awareness in neglect. NeuroImage.

[43] Duprez J, Gulbinaite R, Cohen MX (2020). Midfrontal theta phase coordinates behaviorally relevant brain computations during cognitive control. NeuroImage.

[44] Fassbender C, Hester R, Murphy K, Foxe JJ, Foxe DM, Garavan H (2009). Prefrontal and midline interactions mediating behavioural control. Eur. J. Neurosci..

[45] van Veen V, Carter CS (2002). The anterior cingulate as a conflict monitor: fMRI and ERP studies. Physiol. Behav..

[46] Ullsperger M, von Cramon DY (2001). Subprocesses of performance monitoring: A dissociation of error processing and response competition revealed by event-related fMRI and ERPs. NeuroImage.

[47] Cavanagh JF, Frank MJ (2014). Frontal theta as a mechanism for cognitive control. Trends Cogn. Sci..

[48] Cohen MX (2011). Error-related medial frontal theta activity predicts cingulate-related structural connectivity. NeuroImage.

[49] Cohen MX (2014). A neural microcircuit for cognitive conflict detection and signaling. Trends Neurosci..

[50] Cohen MX, van Gaal S (2014). Subthreshold muscle twitches dissociate oscillatory neural signatures of conflicts from errors. NeuroImage.

[51] McDermott TJ, Wiesman AI, Proskovec AL, Heinrichs-Graham E, Wilson TW (2017). Spatiotemporal oscillatory dynamics of visual selective attention during a flanker task. Neuroimage.

[52] Nolte G, Bai O, Wheaton L, Mari Z, Vorbach S, Hallett M (2004). Identifying true brain interaction from EEG data using the imaginary part of coherency. Clin. Neurophysiol..

[53] Hjorth B (1975). An on-line transformation of EEG scalp potentials into orthogonal source derivations. Electroencephalogr. Clin. Neurophysiol..

[54] Makeig S, Bell AJ, Jung T-P, Ghahremani D, Sejnowski TJ (1997). Blind separation of auditory event-related brain responses into independent components. Proc. Natl. Acad. Sci. USA.

[55] Shaw JC (1981). An introduction to the coherence function and its use in EEG signal analysis. J. Med. Eng. Technol..

[56] Shulman GL, McAvoy MP, Cowan MC, Astafiev SV, Tansy AP, d'Avossa G (2003). Quantitative analysis of attention and detection signals during visual search. J. Neurophysiol..

[57] Brefczynski-Lewis JA, Lutz A, Schaefer HS, Levinson DB, Davidson RJ (2007). Neural correlates of attentional expertise in long-term meditation practitioners. Proc. Natl. Acad. Sci. USA.

[58] Crowe DA, Goodwin SJ, Blackman RK, Sakellaridi S, Sponheim SR, MacDonald AW (2013). Prefrontal neurons transmit signals to parietal neurons that reflect executive control of cognition. Nat. Neurosci..

[59] Goodwin SJ, Blackman RK, Sakellaridi S, Chafee MV (2012). Executive control over cognition: Stronger and earlier rule-based modulation of spatial category signals in prefrontal cortex relative to parietal cortex. J. Neurosci..

[60] Phillips JM, Vinck M, Everling S, Womelsdorf T (2014). A long-range fronto-parietal 5- to 10-Hz network predicts "top-down" controlled guidance in a task-switch paradigm. Cereb. Cortex.

[61] Bartolomeo P, Thiebaut de Schotten M, Chica AB (2012). Brain networks of visuospatial attention and their disruption in visual neglect. Front. Hum. Neurosci..

[62] Eschmann KCJ, Bader R, Mecklinger A (2018). Topographical differences of frontal-midline theta activity reflect functional differences in cognitive control abilities. Brain Cogn..

[63] Cavanagh JF, Cohen MX, Allen JJ (2009). Prelude to and resolution of an error: EEG phase synchrony reveals cognitive control dynamics during action monitoring. J. Neurosci..

[64] Zavala B, Jang A, Trotta M, Lungu CI, Brown P, Zaghloul KA (2018). Cognitive control involves theta power within trials and beta power across trials in the prefrontal-subthalamic network. Brain.

[65] Cabeza R, Ciaramelli E, Olson IR, Moscovitch M (2008). The parietal cortex and episodic memory: An attentional account. Nat. Rev. Neurosci..

[66] Cabeza R, Ciaramelli E, Moscovitch M (2012). Cognitive contributions of the ventral parietal cortex: An integrative theoretical account. Trends Cogn. Sci..

[67] Humphreys GF, Lambon Ralph MA (2017). Mapping domain-selective and counterpointed domain-general higher cognitive functions in the lateral parietal cortex: Evidence from fMRI comparisons of difficulty-varying semantic versus visuo-spatial tasks, and functional connectivity analyses. Cereb. Cortex.

[68] Seghier ML (2013). The angular gyrus: Multiple functions and multiple subdivisions. Neuroscientist.

[69] Gottlieb J, Balan P, Oristaglio J, Suzuki M (2009). Parietal control of attentional guidance: The significance of sensory, motivational and motor factors. Neurobiol. Learn. Mem..

[70] Marzetti L, Di Lanzo C, Zappasodi F, Chella F, Raffone A, Pizzella V (2014). Magnetoencephalographic alpha band connectivity reveals differential default mode network interactions during focused attention and open monitoring meditation. Front. Hum. Neurosci..

[71] Fox KCR, Nijeboer S, Dixon ML, Floman JL, Ellamil M, Rumak SP (2014). Is meditation associated with altered brain structure? A systematic review and meta-analysis of morphometric neuroimaging in meditation practitioners. Neurosci. Biobehav. Rev..

[72] Hölzel BK, Lazar SW, Gard T, Schuman-Olivier Z, Vago DR, Ott U (2011). How does mindfulness meditation work? Proposing mechanisms of action from a conceptual and neural perspective. Perspect. Psychol. Sci..

[73] Kurth F, MacKenzie-Graham A, Toga AW, Luders E (2015). Shifting brain asymmetry: The link between meditation and structural lateralization. Soc. Cogn. Affect. Neurosci..

[74] Luders E, Clark K, Narr KL, Toga AW (2011). Enhanced brain connectivity in long-term meditation practitioners. NeuroImage.

[75] Chica AB, Bartolomeo P, Valero-Cabré A (2011). Dorsal and ventral parietal contributions to spatial orienting in the human brain. J. Neurosci..

[76] Shulman GL, Astafiev SV, McAvoy MP, d'Avossa G, Corbetta M (2007). Right TPJ deactivation during visual search: Functional significance and support for a filter hypothesis. Cereb. Cortex.

[77] Todd JJ, Fougnie D, Marois R (2005). Visual short-term memory load suppresses temporo-parietal junction activity and induces inattentional blindness. Psychol. Sci..

[78] Rounis E, Yarrow K, Rothwell JC (2007). Effects of rTMS conditioning over the fronto-parietal network on motor versus visual attention. J. Cogn. Neurosci..

[79] Rushworth MF, Nixon PD, Renowden S, Wade DT, Passingham RE (1997). The left parietal cortex and motor attention. Neuropsychologia.

[80] Rushworth MF, Krams M, Passingham RE (2001). The attentional role of the left parietal cortex: The distinct lateralization and localization of motor attention in the human brain. J. Cogn. Neurosci..

[81] Rushworth MF, Johansen-Berg H, Göbel SM, Devlin JT (2003). The left parietal and premotor cortices: Motor attention and selection. NeuroImage.

[82] Buschman TJ, Miller EK (2007). Top-down versus bottom-up control of attention in the prefrontal and posterior parietal cortices. Science.

[83] Driver J, Mattingley JB (1998). Parietal neglect and visual awareness. Nat. Neurosci..

[84] Fries P (2015). Rhythms for cognition: Communication through coherence. Neuron.

[85] Gaillard R, Dehaene S, Adam C, Clémenceau S, Hasboun D, Baulac M (2009). Converging intracranial markers of conscious access. PLoS Biol..

[86] Gross J, Schmitz F, Schnitzler I, Kessler K, Shapiro K, Hommel B (2004). Modulation of long-range neural synchrony reflects temporal limitations of visual attention in humans. Proc. Natl. Acad. Sci. USA.

[87] Tang Y, Hölzel BK, Posner MI (2015). The neuroscience of mindfulness meditation. Nat. Rev. Neurosci..

[88] Davidson RJ, Kabat-Zinn J, Schumacher J, Rosenkranz M, Muller D, Santorelli SF (2003). Alterations in brain and immune function produced by mindfulness meditation. Psychosom. Med..

[89] Moyer CA, Donnelly MP, Anderson JC, Valek KC, Huckaby SJ, Wiederholt DA (2011). Frontal electroencephalographic asymmetry associated with positive emotion is produced by very brief meditation training. Psychol. Sci..

[90] Luu P, Flaisch T, Tucker DM (2000). Medial frontal cortex in action monitoring. J. Neurosci..

[91] Yordanova J, Falkenstein M, Hohnsbein J, Kolev V (2004). Parallel systems of error processing in the brain. NeuroImage.

[92] Tomita H, Ohbayashi M, Nakahara K, Hasegawa I, Miyashita Y (1999). Top-down signal from prefrontal cortex in executive control of memory retrieval. Nature.

[93] Beauregard M, Levesque J, Bourgouin P (2001). Neural correlates of conscious self-regulation of emotion. J. Neurosci..

[94] Kerns JG, Cohen JD, MacDonald AW, Cho RY, Stenger VA, Carter CS (2004). Anterior cingulate conflict monitoring and adjustments in control. Science.

[95] Levesque J, Eugène F, Joanette Y, Paquette V, Mensour B, Beaudoin G (2003). Neural circuitry underlying voluntary suppression of sadness. Biol. Psychiatry..

[96] Shamay-Tsoory SG, Tomer R, Berger BD, Aharon-Peretz J (2003). Characterization of empathy deficits following prefrontal brain damage: The role of the right ventromedial prefrontal cortex. J. Cogn. Neurosci..

