# Supplementary material for: Attentional and cognitive monitoring brain networks in long-term meditators depend on meditation states and expertise
Source: Sci Rep. 2021 Mar 1;11:4909. doi: 10.1038/s41598-021-84325-3 (PMC7921394; doi:10.1038/s41598-021-84325-3)
Supplement: Supplementary file 1 — Supplementary Information. [file 41598_2021_84325_MOESM1_ESM.pdf]

# Attentional and cognitive monitoring brain networks in long-term meditators depend on meditation states and expertise

Juliana Yordanova[1], Vasil Kolev[1], Valentina Nicolardi[2,3], Luca Simione[4], Federica Mauro[2], Patrizia Garberi[2], Antonino Raffone[2,5], & Peter Malinowski[6]

[1] Institute of Neurobiology, Bulgarian Academy of Sciences, Sofia, Bulgaria
[2] Department of Psychology, Sapienza University of Rome, Italy
[3] Social and Cognitive Neurosciences Laboratory, IRCCS, Santa Lucia Foundation, Rome
[4] Institute of Cognitive Sciences and Technologies, CNR, Rome, Italy
[5] School of Buddhist Studies, Philosophy and Comparative Religions, Nalanda University, Rajgir, India
[6] School of Psychology, Research Centre for Brain and Behaviour, Liverpool John Moores University (LJMU), Liverpool, UK

## SUPPLEMENTARY INFORMATION

Highly experienced "virtuoso" meditators as targeted in the present study are typically experts in different styles of meditation, and in the context of any tradition, they are not trained in only one particular meditation style. Also, the current experimental design included a fixed order of three meditation styles. We tested if the common involvement of left posterior theta FP and MF networks may reflect a generalized network activity induced by the integrated meditation training of participants or carry over effects from one meditation state to another in the current experimental design. With this regard, the following hypotheses were tested.

### Hypothesis 1. "A generalized network activity might reflect a generalized form of meditation training"

According to the main results presented in the paper, for all meditation styles, the left parietal nodes of the FP *theta* networks exhibited increased synchronization with frontal regions in the same (left) hemisphere, and the left parieto-occipital regions manifested increased synchronization with the medial frontal cortex within the MF network. It is important to be emphasized that these observations were based on analyses of subtracted values (rest minus meditation), meaning that the three forms of meditation induced ***common patterns of change*** relative to the resting state. However, it may still be possible that a generalized form of meditation training would correspond to a common connectivity pattern that is shared by all three meditation styles rather than to a common pattern of change that would reflect a shared involvement of executive systems (as interpreted in the paper).

To check for the existence of such a generalized network we performed analyses, in which we used the original (un-subtracted) *ICoh* values in the theta band, with a special focus on connectivity characteristics of left parietal FP nodes lobes and MF connections of the medial frontal areas with left parietal and parieto-occipital lobes. Analysis design for the FP networks included 3 within-subjects variables: *Meditation Condition* (REST vs. FAM vs. OMM vs. LKM), *Frontal nodes* (F5, F3, F4, F5), and *Parietal nodes* (P5, P3, P4, P6). Analysis design

for the MF network also included the within variable *Meditation Condition* and relevant *Region* (6 levels) and *Laterality* (5 levels) topography factors, as described in the manuscript.

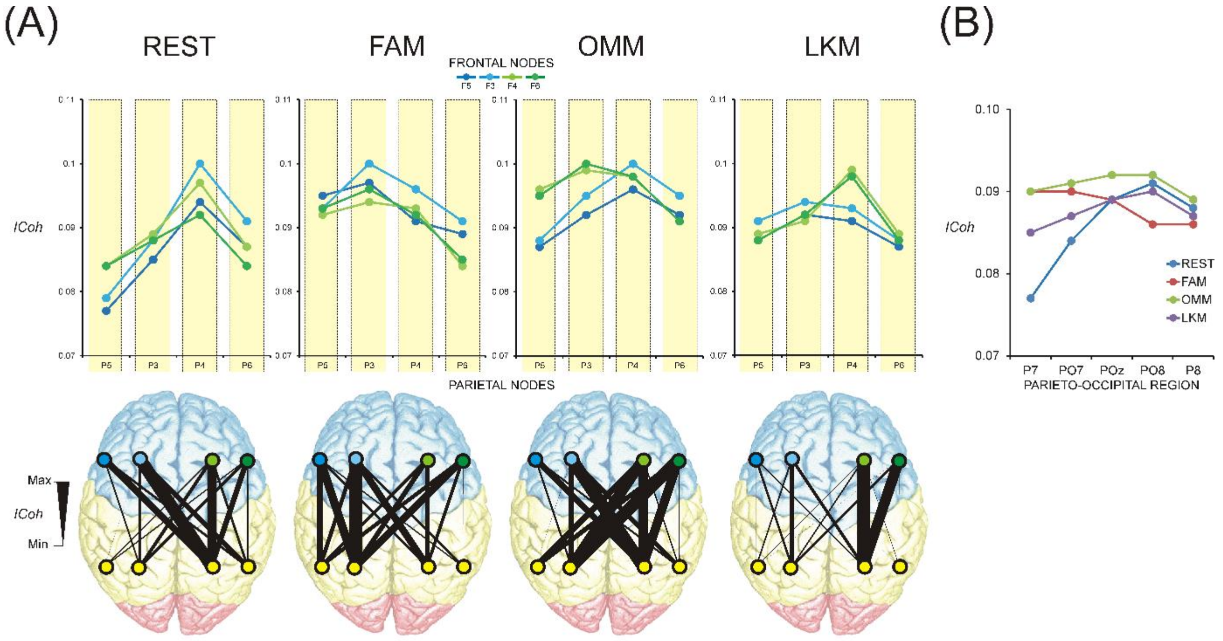


**Figure 1.** (A) Connectivity of fronto-parietal networks during 4 conditions – REST, FAM, OMM, LKM. In the upper panel, Group means of original *ICoh* values in the theta frequency band (4-7 Hz) are presented for each fronto-parietal electrode pair, e.g., P5-F5, P5-F3, P5-F4, P5-F6, etc. Parietal nodes P5, P3, P4 and P6 are marked in yellow, whereas the frontal nodes are in different colours as indicated in the figure (F5, F3, F4, F6). In the lower panel, *ICoh* values in each condition are presented graphically after being coded by the thickness of lines. (B) Connectivity of medial fronto-parieto-occipital networks during 4 conditions – REST, FAM, OMM, LKM in the theta band.

Figure 1A demonstrates that in experienced meditators during REST, the right dorsal parietal node (P4) of FP networks manifested maximal connectivity with each of the frontal nodes. In contrast, during FAM, left parietal nodes (P5 and P3) were more strongly connected with each frontal node as compared to the right parietal nodes, whereas during OMM, the strongest connections were inter-hemispheric - between left parietal and right frontal nodes, and between right parietal and left frontal nodes. LKM appeared to have expressed predominant intra-hemispheric dorsal FP connections in the right hemisphere. Although these observations were partially supported by the tangential significance of the interaction *Meditation Condition* x *Parietal nodes*, $F(9/189) = 2.23$, $p = 0.08$), they were not reported in the paper because the statistical outcome did not reach validity to justify their presentation (*Meditation Condition* x *Frontal nodes* ($F(9/189) = 1.08$, $p = 0.4$; *Meditation Condition* x *Frontal nodes* x *Parietal nodes*, $F(27/567) = 1.5$, $p = 0.2$).

Likewise, Fig. 1B demonstrates that the connections of theta MF network with parietal and parieto-occipital lobes were prominently stronger in the right hemisphere during REST and LKM, in the left hemisphere during FAM, without clear lateralisation patterns during OMM. As observed for FP networks, these results were not statistically significant (*Meditation Condition* x *Region* x *Laterality*, $F(60/1200) = 1.52$, $p = 0.14$), and respectively, not included in the paper.

Although non-significant, these observations provide some evidence that despite the integrated training of participants, and despite the integrated experimental session including

the three meditation styles, the connectivity patterns of FP and MF networks are distinct across meditation states. Therefore, a generalized network may not be responsible for the specific left-lateralised increase in FP and MF connectivity common for all states of mediation. Rather, an enhanced involvement of left-lateralised nodes, irrespective of style-specific network properties, appears as a common activation induced by any type of meditation, as interpreted in the study.

### Hypothesis 2. "Contrasts of resting and meditation states might be influenced by trait-like characteristics of the expert mediation practitioners and by spontaneously establishing states of mindfulness even at rest"

Long-term meditation training may induce neuroplastic alterations in executive and cognitive networks which may produce a spontaneous change in the activity of these networks even at rest, accompanied by the associated change in the mental state. Within this hypothesis, we use the analyses of un-subtracted values presented above to demonstrate that the state of REST in experienced meditators is characterized by dominating connectivity of right-hemisphere dorsal nodes of FP and FM networks. This connectivity pattern is not identical to the ones characterizing FAM, OMM or LKM. Hence, even if the connectivity pattern during REST may be altered by neuroplastic reorganization of executive networks, the corresponding mental state during rest may not be identical to a mental state sustained during FAM, OMM or LKM.

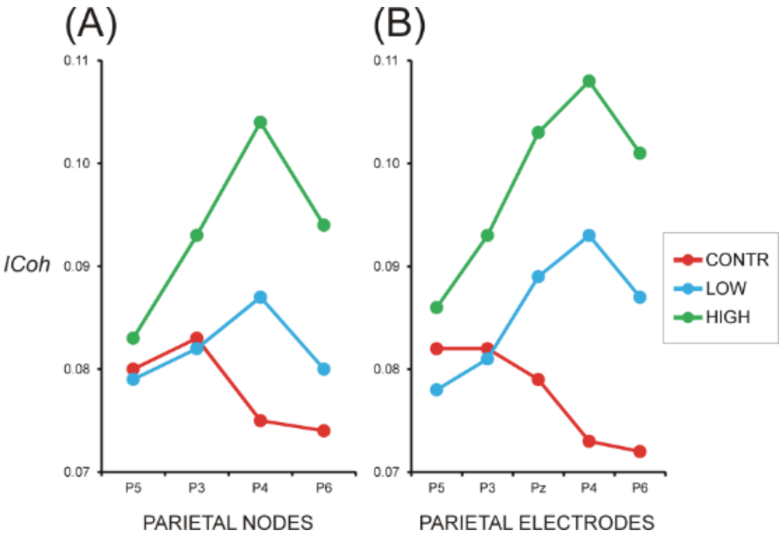


**Figure 2.** Connectivity of the parietal nodes of FP networks (A) and FM networks (B) during REST in three groups of participants – high-level experts, low-level experts, and non-experts (controls). Group means of original *ICoh* values in the theta frequency band (4-7 Hz) are presented.

We also tested if FP and MF connections during REST might have been responsible for the linear and non-linear dependencies on *Meditation Expertise* observed for subtracted values. Original *ICoh* values of FP and MF connections during REST were included in linear and quadratic regression models. In contrast to significant dependencies found for subtracted values as reported in the paper, no model solution determined a significant prediction of *Meditation Expertise* for unsubtracted *ICoh* measures during REST.

Even though, the presumed expertise-dependent neuroplastic changes in connectivity during REST may have produced or affected the results about meditation effects obtained with subtracted values. We performed two tests in this direction. First, using the median of practice duration (*Meditation Expertise*), the group of expert meditators was split into two subgroups, comprising low-level and high-level experts (n = 11 in each group) with mean duration of practice of 7455 hours (SD = 4736) and 19358 hours (SD = 12275), respectively. The effects

of *Group* (low- vs. high-level experts) were tested in a *Group* x *Frontal Nodes* x *Parietal Nodes* ANOVA (for FP networks) and *Group* x *Region* x *Laterality* (for MF network) during REST. It was expected that if FP and MF networks during REST were differentially modified by neuroplastic changes due to the level of expertise, significant main and interactive effects of *Group* would be yielded. The results demonstrated that none of the main or interactive effects of the *Group* factor were significant ($p > 0.1$). Figure 2A shows that low-level experts indeed manifested weaker connectivity during REST, but this difference was not significant (*Group*, $F(1/20) = 2.7$, $p = 0.12$). The strongest connectivity of the right dorsal parietal node with frontal nodes was statistically verified (*Parietal nodes*, $F(3/60) = 6.24$, $p = 0.01$). More importantly, however, the right dorsal parietal node of FP networks was most strongly connected with frontal nodes in each of the two sub-groups. Accordingly, the interactions *Group x Parietal nodes*, $F(3/60) = 1.3$, $p = 0.3$ and *Group x Frontal nodes x Parietal nodes*, $F(9/180) = 1.7$, $p = 0.2$, were not significant. The same results were yielded for MF network analysis during rest where no significant main or interactive effects of *Group* were found ($p > 0.3$) – Fig. 2B.

Including non-experts (Controls, n = 11) in the analysis confirmed statistically the presence of expertise-dependent increase in FP connectivity during REST (*Group*, $F(2/30) = 3.22$, $p = 0.05$). Moreover, this analysis specified that the main expertise-dependent change in FP networks during REST involved the ***parietal nodes in the right***, but not in the left hemisphere (*Group x Parietal nodes*, $F(6/90) = 3.1$, $p = 0.04$; *Group* effects for P4 and P6 connections with any frontal node, $F(2/30) = 3.3 \div 5.9$, $p = 0.05 \div 0.007$; *Group* effects for P3 and P5 connections, $F(2/30) < 1.2$, $p > 0.3$) – Fig. 2A. Likewise, only right-hemisphere posterior connections with medial frontal cortex increased with expertise increase ($F(2/30) = 3.3 \div 7.7$, $p = 0.05 \div 0.002$) – Fig. 2B.

These observations indicate that the FP and MF connections appear to be strengthened with expertise during REST. However, control analyses during REST show that (1) no significant differences exist between the groups of low- and high-level experts, (2) major expertise-related alterations emerge for right but not left-hemisphere connections which were sensitive to states of meditation, (3) the major patterns of FP and MF connectivity during REST are similar in low- and high-experts, irrespective of expertise level.

**Hypothesis 3. "Generalized network activity observed across meditation styles might be specific to the experiment design, which might have afforded carry-over effects from one meditation state to the next or not necessarily require participants to change from one state to another"**

Analyses of original *ICoh* values presented above showed that the connectivity patterns of FP and MF networks were distinct across meditation states despite the integrated experimental design including the three meditation styles. If the fixed order of the meditation states induced a generalization of FP and MF networks activation, no specific connectivity patterns would emerge, and these patterns would be similar across conditions. The observation that these patterns were distinctive implies that FP and MF networks are involved in specific ways in each meditation condition, and carry over effects may not be responsible for the reported meditation-related commonalities in the activation of left posterior nodes of these networks.

We performed analysis of connectivity in different states (REST, FAM, OMM, LKM) maintained during Session 1 and Session 2 of the experiment. We hypothesized that if the fixed order of mental states used in the current experimental design were the main factor for connectivity increase observed as a common feature of the three meditation styles, (1) left

posterior connectivity increase would be progressively emphasized from the first to the third meditation state (from FAM to LKM), (2) left posterior connectivity enhancement of "generalization" would be more expressed in the second than in the first session, (3) critically, this effect would affect also the REST state in the second session following the three meditation styles in the previous first session. The factor *Session* (first vs. second) was used as a within-subjects factor in the *Meditation Condition x Session* ANOVA of left parietal nodes connections.

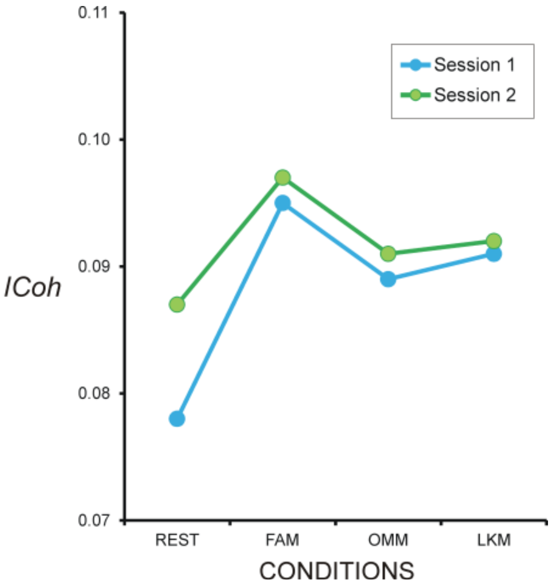


**Figure 3.** Connectivity of the left parietal nodes of FP networks during four conditions REST, FAM, OMM, and LKM in 2 consecutive experimental sessions (Session 1 and Session 2). Group means of original *ICoh* values in the theta frequency band (4-7 Hz) are presented for the group of experienced meditators (n = 22).

Analysis of left parietal FP nodes yielded a significant effect of *Meditation Condition* ($F(3/63) = 2.9$, $p = 0.05$) reflecting the meditation-related connectivity increase relative to REST (Fig. 3). However, the effects of *Session* ($F(1/21) = 1.7$, $p = 0.2$) and *Session x Meditation Condition* ($F3/63) = 0.25$, $p = 0.8$) were not significant. As demonstrated in Fig. 3, the relationships among left posterior connectivity in different states were preserved in the two sessions, disconfirming the possibility of smeared or unified left posterior patterns. The same effects were obtained in the analysis of left posterior regions of the MF network (*Session*, $F(1/21) = 1.2$, $p = 0.3$; *Session x Meditation Condition*, $F(3/63) = 1.9$, $p = 0.15$). These results imply that the design with fixed order of meditation states in two consecutive sessions employed in the present study might not be responsible for the shared enhancement of left posterior connectivity of FP and MF networks.
